# Changes in Glycemic Control Following Use of a Spanish-Language, Culturally Adapted Diabetes Program: Retrospective Study

**DOI:** 10.2196/40278

**Published:** 2022-12-07

**Authors:** Caitlyn Edwards, Elisa Orellana, Kelly Rawlings, Mirta Rodriguez-Pla, Aarathi Venkatesan

**Affiliations:** 1 Vida Health San Francisco, CA United States

**Keywords:** type 2 diabetes, digital health, diabetes intervention, diabetes, mobile health, mhealth, app-based, health coaching, HbA1c, glycemic improvements, localization, Spanish, health application, health education, patient education, nutrition, digital health intervention, health management

## Abstract

**Background:**

Several barriers to diabetes treatment and care exist, particularly in underserved medical communities.

**Objective:**

This study aimed to evaluate a novel, culturally adapted, Spanish-language mHealth diabetes program for glycemic control.

**Methods:**

Professional Spanish translators, linguists, and providers localized the entirety of the Vida Health Diabetes Management Program into a culturally relevant Spanish-language version. The Spanish-language Vida Health Diabetes Management Program was used by 182 (n=119 women) Spanish-speaking adults with diabetes. This app-based program provided access to culturally adapted educational content on diabetes self-management, one-on-one remote counseling and coaching sessions, and on-demand in-app messaging with bilingual (Spanish and English) certified health coaches, registered dietitian nutritionists, and certified diabetes care and education specialists. Hemoglobin A_1c_ (HbA_1c_) was the primary outcome measure, and a 2-tailed, paired *t* test was used to evaluate changes in HbA_1c_ before and after program use. To determine the relationship between program engagement and changes in glycemic control, a cluster-robust multiple regression analysis was employed.

**Results:**

We observed a significant decrease in HbA_1c_ of –1.23 points between baseline (mean 9.65%, SD 1.56%) and follow-up (mean 8.42%, SD 1.44%; *P*<.001). Additionally, we observed a greater decrease in HbA_1c_ among participants with high program engagement (high engagement: –1.59%, SD 1.97%; low engagement: –0.84%, SD 1.64%; *P*<.001).

**Conclusions:**

This work highlights improvements in glycemic control that were clinically as well as statistically significant among Spanish-preferring adults enrolled in the Vida Health Spanish Diabetes Management Program. Greater improvements in glycemic control were observed among participants with higher program engagement. These results provide needed support for the use of digital health interventions to promote meaningful improvements in glycemic control in a medically underserved community.

## Introduction

Diabetes impacts more than 37.3 million, or 11.3%, of all people in the United States [1]. Attributable to a combination of genetic, cultural, and socioeconomic factors, Hispanic/Latino Americans are more likely to have type 2 diabetes (17%) compared to non-Hispanic White Americans (8%), are more likely to develop diabetes across their lifespan, and are more likely to develop diabetes at a younger age [2,3].

Clinical guidelines recommend that individuals diagnosed with diabetes achieve and subsequently maintain a hemoglobin A_1c_ (HbA_1c_) level of ≤7%. Prospective studies, such as the United Kingdom Prospective Diabetes Study and the Diabetes and Aging Study, have identified associations between a higher level of HbA_1c_ in individuals with diabetes and an increased risk of both micro- and macrovascular health events [4,5]. Clinical guidelines for diabetes treatment therefore center around successful glycemic management achieved through behavior changes, including long-term self-monitoring of dietary intake and physical exertion, and in some cases, blood glucose, as well as other behaviors, such as taking medicine as directed, obtaining biometric-based feedback, and participating in high levels of provider and patient interaction. These behavior changes are encompassed and defined as diabetes self-management education and support (DSMES) [6]. Many structural and personal barriers to treatment exist, and effective diabetes management can be difficult for both patients and providers [7]. Lack of appointment availability, provider access, time, finances, and the influence of other co-occurring health conditions are just a few of the obstacles to meeting clinical guidelines and reducing individual disease burden.

Hispanic/Latino Americans may face additional barriers to seeking treatment that are directly related to communication and culture [7]. Accessing providers who speak their language and understand their cultural values, preferences, and approaches to health is an additional challenge for many communities, resulting in medically underserved communities in which individuals are not able to seek, implement, or sustain the recommendations of DSMES [8] and essential self-care behaviors [9]. Indeed, research suggests that many DSMES interventions in usual care settings are considerably less successful in patients from ethnic minority groups compared to their White counterparts, with worse outcomes, lower rates of participation, and higher attrition rates [10]. There have been robust findings of large gaps in the present diabetes treatment literature and the health care community as a whole, and there is a need for an evidence-based understanding of the unique needs of medically underserved communities. The development and subsequent evaluation of personalized and language-concordant interventions is needed.

Existing work examining personalized care addressing underserved populations confirms a prolonged decrease in HbA_1c_ when health care is used that is culturally adapted; in other words, when health care is used that is adapted to relevant culture-based food and eating patterns, familial hierarchies, or culture-based beliefs or knowledge about diabetes [11]. A recent Cochrane systematic review and meta-analysis found that culturally appropriate diabetes health education in ethnic minority groups can result in significant improvements in HbA_1c_, triglycerides, and knowledge about diabetes and its management [8]. Additionally, improvements in HbA_1c_ were found to be higher with culturally appropriate health education interventions compared to “usual care” interventions, and these results were sustained for up to 2 years following the studied interventions [8]. One potential mechanism of these improvements in outcomes is the reduction of language discordance between providers and patients with limited English proficiency (LEP). LEP has been associated with reports of suboptimal interactions between providers and patients with diabetes, and the prevalence of glycemic control has been shown to increase among Latinos with LEP who switched care from a language-discordant provider to a language-concordant provider [12,13]. This previous work highlights the achievability and potential effectiveness of the development and implementation of culturally appropriate interventions and suggests that health care organizations should make efforts toward implementing such interventions.

One proposed approach to increasing the implementation of such programs is the use of mobile health (mHealth) interventions. The concept of mHealth is broadly used to describe smartphone-, tablet-, or personal computer–based health care [14]. Interventions that use mHealth are highly customizable and have immense potential for localization, which is the “adaptation of a product, application, or document content to meet the language, cultural, and other requirements of a specific target market” [15]. Due to this, mHealth interventions for diabetes treatment are becoming more commonplace and have been shown to be effective in both improving glycemic control and providing cost savings for English-speaking participants [14,16]. Surprisingly, despite widespread usage of smartphones among the Hispanic/Latino community [17], as well as evidence of successful technology-based behavior change interventions, mHealth diabetes programs localized for use in the Hispanic/Latino community are lacking [18]. Of all diabetes apps offered on app stores, only 41% of Android apps and 21% of iOS apps are available in Spanish. Additionally, most apps offering Spanish-language content are written at a complex (ie, >fifth grade) reading level or do not provide their user interface in Spanish [19]. This a concerning missed opportunity for providing equitable care, as properly designed mHealth provides a unique ability to increase access not only to cost-effective and easily accessible evidence-based diabetes care, but also to culturally appropriate, personalized, and language-concordant care and providers.

In response to this gap in the field of diabetes treatment and management, the Vida Health Diabetes Management Program was localized as the Vida Health Spanish Diabetes Management Program. The Vida Health Diabetes Management Program is an app-based digital health platform for the treatment and management of chronic diseases. The primary aim of this study was to evaluate the effectiveness of the Vida Health Spanish Diabetes Management Program on measures of glycemic control (ie, HbA_1c_) among a cohort of Spanish-preferring adults from the United States. The secondary aim was to quantify and examine engagement metrics of the Vida Health Spanish Diabetes Management Program and its potential impact on glycemic control.

## Methods

### Ethical Considerations

This study was approved by an independent institutional review board (Western Institutional Review Board), which waived informed consent; the study was identified as having minimal risk because the data were fully anonymized before use in the analysis.

### Recruitment and Enrollment

Adults from a single major-payer client of Vida Health were recruited for this study through brochures, calling campaigns, and email announcements. Potential participants were offered the app-based program as part of their medical insurance benefits. All participants enrolled in the study had smartphone- or web-based access. Participants accessed the program through the Apple App Store or the Google Play Store and entered an invitation code unique to their insurance carrier for complementary program usage. The program is offered in both English and Spanish; the language used is based on the default language preference of the participants’ mobile phone. All subsequent engagement with the Vida Health app refers to the translated and adapted Spanish-language app interface and content.

After app installation, participants completed a number of internally designed intake forms, where they provided contact information for app notifications, basic demographic information including age and sex, and self-reported existing health conditions. Participants were excluded from study participation if they indicated the presence of type 1 diabetes, stage 4 or 5 chronic kidney disease, or class III or IV congestive heart failure; were pregnant; or were breastfeeding.

Laboratory data pertaining to glycemic control (ie, HbA_1c_ level) was provided by the insurance carrier at a monthly cadence using protected and standardized data-sharing arrangements.

### Vida Health Spanish Diabetes Management Program

The Vida Health Diabetes Management Program is an app-based intervention designed to enable and empower individuals to manage their health through frequent provider interaction and rigorously designed multimedia content. Providers offered by the program include bilingual (Spanish and English) certified health coaches, registered dietitian nutritionists, and certified diabetes care and education specialists. Professional Spanish translators and linguists, as well as bilingual providers, localized the entirety of the Vida Health Diabetes Management Program into a culturally relevant Spanish-language version. Localization, as defined above, differs from translation. An example of localization, as opposed to mere translation, is how Vida approaches nutrition education content in the app. A translation approach entails term replacement, for example, swapping the term “1 rodaja pan” for “1 slice bread” in a lesson about using the Healthy Plate meal-planning approach at breakfast. The localization method looks at term use with a cultural and accessibility lens. An example of this includes providing a culturally equivalent recipe (eg, a favorite holiday dish traditionally made in Spanish-speaking countries instead of a more United States–influenced holiday special). Localization also affects lists of ingredients on labels. Alongside the Spanish translation of a word, the English-language version is shown, to match what any person living in the United States would find at a grocery store (eg, “whole-grain flour”). If participants had any issue logging food or recipes in the app, they were encouraged to reach out to their provider for clarity or to send a picture of their meal to their provider, who could assist with logging.

After completing app intake, participants were paired with a bilingual (Spanish and English) certified health coach, a registered dietitian nutritionist, a certified diabetes care and education specialist, or a combination of these providers. To account for licensure requirements and cultural differences in geographic locations, providers were matched to participants by their state of residence. All providers received extensive evidence-based training on diabetes self-management and operated under a motivational interviewing framework to promote self-efficacy and autonomy for behavior change [20]. Motivational interviewing is an evidence-based skill set designed to aid individuals in identifying motivation for change and to facilitate personalized changes in lifestyle and behavior [21]. To provide support for bilingual providers, all training was available in both Spanish and English. Additional training was provided on the nutrition guidelines of various Spanish-speaking countries.

**Figure 1:**
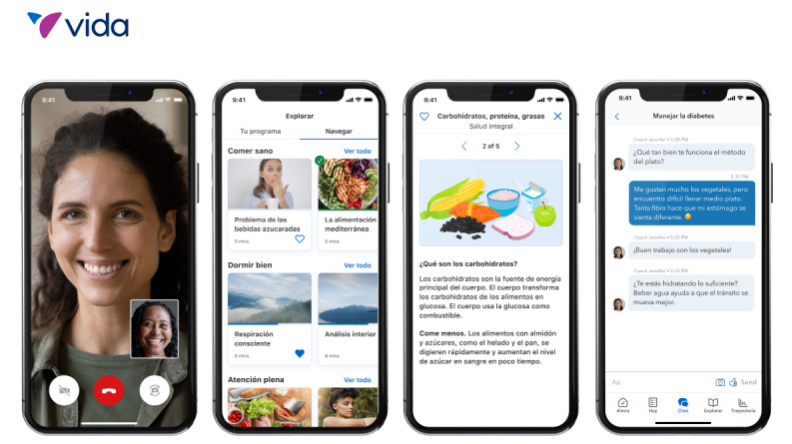
Screenshots of the Vida Health Spanish Diabetes Management Program.

Full information on the program is described in previously published work [22]. Briefly, the program was designed from both the provider and participant perspectives to operationalize a complete feedback loop. The first interaction between provider and participant included a detailed assessment to identify personalized participant health goals and potential barriers to outcome success. All providers had the option to complete internal and external communications and notes in Spanish to prevent the need for switching between languages during consultations. Follow-up provider support was provided through on-demand in-app messaging and remote video sessions (the videos sessions were weekly for the first 12 weeks and monthly thereafter), in which providers and participants worked to follow up on goal progress and resolve ambivalence to change. Between provider sessions, participants were encouraged to message their providers as often as needed and received daily in-app content to provide education and activation opportunities and to support treatment goals (Figure 1). All app usage was tracked by the providers, and biometric data was collected at each video session to allow for up-to-date data monitoring, interpretation, and pertinent adjustment of treatment as needed. Participants were additionally encouraged to reach out to their providers for app support if any issues or questions arose regarding the app.

Daily app content included evidence-based structured lessons and multimedia content pertaining to nutrition, taking medicine, self-monitoring, and other behaviors outlined in the ADCES (Association of Diabetes Care and Education Specialists) 7 Self-Care Behaviors [9,23]. Alongside app-generated content, participants are encouraged to engage in a variety of data-logging activities within the program app: food logging, physical activity logging, and self-monitoring of blood glucose when appropriate. The Vida Health food logging system included a comprehensive list of culturally relevant food selections for various Spanish-speaking countries, allowing for accessible food logging. Additionally, providers were trained in relevant cultural and regional clinical nutrition guidelines to ensure informed care was provided that considered an individual’s unique traditions, customs, and values. This training and these potential resources included continuing education and reference documentation on country- or region-specific staple foods and their corresponding nutrient breakdown, common traditions and celebrations, and customs surrounding meal timing. Screenshots of the Vida Health user interface are provided in Figure 1.

### Statistical Analysis

All data preparation and analyses were conducted in Python (version 3.7.8) and R (version 4.1.2, R Studio Team). The primary outcome measure for this study was HbA_1c_. HbA_1c_ was measured in clinical laboratories; the data were made available by the program’s client. Baseline HbA_1c_ was defined according to the laboratory test results that were closest to the program start, measured between 1 year before (–365 days) to within 21 days after enrollment. Follow-up HbA_1c_ was defined as the most recent HbA_1c_ assessment within 12 months of enrollment and at least 90 days from the program start. A 2-tailed, paired *t* test was used to assess changes in HbA_1c_ from baseline.

Program engagement was a secondary focus of this study. First, we computed a cumulative sum for each of the following factors: (1) messages sent between a participant and their provider, (2) the number of consultations with a provider completed by a participant, and (3) the amount of curriculum content completed within the first 12 weeks (the most intensive phase of the intervention) of the program start. Then, we created a binary engagement variable, in which “high engagement” was defined as engagement for each of our 3 defining engagement metrics that was greater than or equal to the median. A cluster-robust multiple regression analysis using the participants as a clustering factor was used to evaluate the association of level of engagement with HbA_1c_ change.

## Results

### Sample Demographics

Baseline HbA_1c_ data were available for 354 individuals. Follow-up HbA_1c_ data were available for 182 individuals. Full demographics for the final study cohort are shown in Table 1.

**Table 1:** Demographic and engagement information for a cohort of Spanish-preferring individuals enrolled in a Spanish-language mHealth intervention (N=182).

Characteristics			Value
**Sex^a^, n**			
	Female	119	
	Male	59	
Baseline HbA_1c_^b^ (%), mean (SD)			9.65 (1.56)
Follow-up HbA_1c_ (%), mean (SD)			8.42 (1.44)
Age (years), mean (SD)			55.67 (8.91)
**Age category (years), n (%)**			
	20 to 40	11 (6)	
	40 to 60	119 (65.4)	
	Older than 60	52 (28.6)	
Messages sent, mean (SD)			27.72 (44.26)
Consultations completed, mean (SD)			8.00 (7.63)
Curriculum completed, mean (SD)			8.95 (16.38)

^a^Four individuals did not provide data for this category.

^b^HbA_1c_: hemoglobin A_1c_.

### Primary Outcome

**Figure 2:**
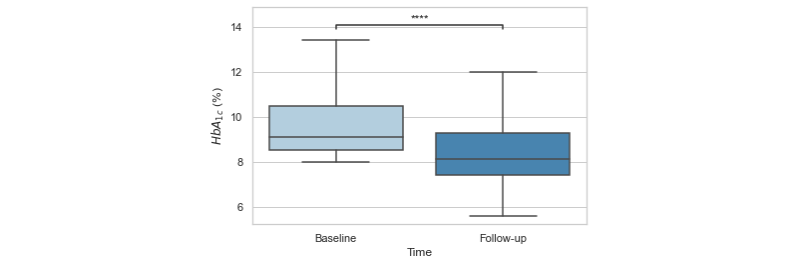
Box plot showing significant differences between HbA_1c_ at baseline and at a minimum 90-day follow-up in a sample of Spanish-preferring (N=182) individuals enrolled in the Vida Health Spanish Diabetes Management Program. HbA_1c_: hemoglobin A_1c_.

Baseline HbA_1c_ measurements were completed on average –56.70 (SD 67.49) days prior to the program start, and follow-up HbA_1c_ measurements were completed on average 157.94 (SD 54.38) days from the program start. A paired *t* test revealed a significant reduction in HbA_1c_ of –1.23 percentage points between baseline (mean 9.65%, SD 1.56%) and follow-up (mean 8.42%, SD 1.44%; *t_181_*=–8.99; *P*<.001; Figure 2).

### Program Use Outcomes

**Figure 3:**
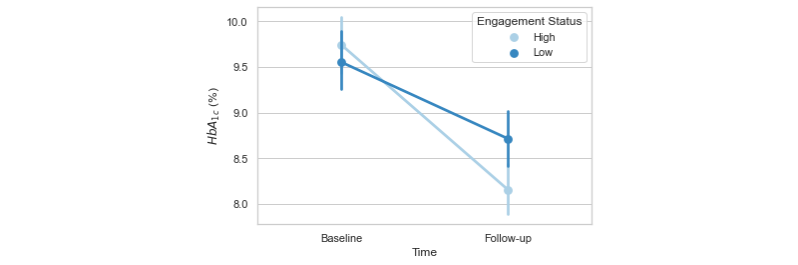
Baseline and follow-up HbA_1c_ dichotomized by a median split of intervention engagement into “high” and “low” engagement categories (N=182). HbA_1c_: hemoglobin A_1c_.

Program engagement data was available from all 182 individuals. Engagement metrics for up to 3 months (12 weeks) following program enrollment were calculated for (1) messages sent between a participant and their provider, (2) the number of consultations with their provider completed by a participant, and (3) the amount of the curriculum content completed. The engagement data were heavily leftward skewed, indicating the presence of a group of high program users. To account for this, the median of each engagement metric was used to dichotomize participants into “high” (95/182, 52.2%) or “low” (87/182, 47.8%) app users. No differences in age (*P*=.16) or baseline HbA_1c_ (*P*=.41) were observed between these engagement groups. To determine potential relationships between app engagement and glycemic control, a cluster-robust multiple regression analysis was conducted using participant as a clustering variable. This allowed the inherent standard error between providers to cluster on the participant. Age, sex, and the binary high or low engagement group status were included as fixed factors, and baseline HbA_1c_ was included as a covariate. Age (*P*=.42) and sex (*P*=.89) were not found to be predictive of a change in HbA_1c_ at follow-up, while baseline HbA_1c_ value was predictive of a change in HbA_1c_ (β=–.77, *P*<.001). A main effect of engagement status was observed (β=–.31, *P*=.003), in which high program users exhibited a higher change in HbA_1c_ over the course of the intervention than did low program users (high group mean change was –1.59%, SD 1.97%; low group mean change was –0.84%, SD 1.64; Figure 3).

## Discussion

### Principal Findings

This study aimed to evaluate the effectiveness of a localized Spanish-language diabetes management program for improving measures of glycemic control among a cohort of Spanish-preferring adults from the United States. We demonstrated that in a cohort of Spanish-preferring individuals with diabetes, enrollment in the Vida Health Spanish Diabetes Management Program was effective in significantly improving glycemic control. We also found that individuals with higher intervention engagement demonstrated higher improvements in glycemic control than individuals with lower intervention engagement.

Many barriers exist to diabetes treatment, and these barriers are only exacerbated in medically underserved communities [7]. The goal of developing the Vida Health Spanish Diabetes Management Program was to reduce barriers to diabetes education, diabetes self-management tools, and diabetes care provider access. The large effect on HbA_1c_ (–1.23 percentage points) observed in this study lends support to the belief that comprehensively designed, culturally adapted mHealth interventions can result in clinically significant health benefits. Additionally, this result is consistent in magnitude with findings from the non-Spanish Vida Health Diabetes Management Program, and the decrease we observed was larger than the 0.1% to 0.7% reduction found in a recent Cochrane review of the potential benefits for glycemic control of culturally appropriate diabetes interventions [8,22]. These findings indicate a significant clinical impact on the study participants, as an improvement in HbA_1c_ greater than 1% over 10 years has been associated with a 21% reduction in diabetes-related deaths and a 37% reduction in microvascular complications [5,24]. A reduction of over 1% was achieved as early as 90 days following program enrollment, potentially allowing for the “legacy effect” of the physiological benefits of HbA_1c_ reduction to begin earlier and be enjoyed for longer across the lifespan.

### Comparison to Prior Work

Our secondary aim explored the hypothesis that participants with higher engagement with the program would see greater improvements in glycemic control, as has been demonstrated in both the non-Spanish Vida Health Diabetes Management Program [22] and other culturally adapted diabetes education programs [16]. The preferred type of engagement with programs designed to increase self-management of type 2 diabetes has been shown to vary, with participants expressing differing preferences for different design features [25]. Therefore, a composite measure of engagement was used to capture program engagement across potential individual-level differences in preferred methodology of engagement. While both high- and low-engagement participants demonstrated improvements in HbA_1c_, we found that higher-engaging individuals, who completed more sessions with their providers, sent more messages to their provider team and completed more of the program curriculum; they also saw an improvement in glycemic control that was –0.75 percentage points greater than that seen by the lower-engaging participants. Achievement of this level of engagement was possible because this study is among the first, to the authors’ knowledge, to use a smartphone app for a localized Spanish-language diabetes mHealth intervention. Recent advancements in smartphone technology have facilitated enhanced capabilities for mHealth interventions, allowing for app engagement opportunities not feasible in previous trials. While much work has been conducted in English-preferring populations to expand health-based interventions, the same level of expansion has not been applied to Spanish-preferring populations. As an example, the previous TExT-MED trial found trend-level improvements in HbA_1c_ in individuals with diabetes who participated in a unidirectional text-message intervention compared to those who completed a usual care intervention [26]. Had this, and other previous work, been able to expand upon text messaging with other smartphone-enabled technology to support localized and culturally adapted content, it is possible significant results would have been observed. The ability to mimic in-person “gold standard” diabetes treatments, alongside the enhanced ease of access due to the ubiquity of technology and smartphone use, allows for comprehensive and bidirectional diabetes care in typically medically underserved communities. While future work is needed to explore potential factors associated with being a high- or low-engagement participant, what we can conclude is that the Vida Health Spanish Diabetes Management Program provides an effective and accessible way of providing diabetes care for those who engage with its content and experience.

It is also possible that participants who had higher engagement were more motivated to engage in and manage their health, as motivation to maintain a healthy lifestyle is key to diabetes management [27]. While motivation was not directly measured in the current work, the Vida Health Spanish Diabetes Management Program removed many obstacles to care that may have impeded participants’ motivation to engage in diabetes treatment. Glazier et al [28] examined strategies to improve the response to cultural interventions in individuals with diabetes and found that most successful interventions used cultural tailoring of the intervention, provided more than 10 contacts between the individual and the intervention, provided a longer intervention duration than typical didactic education methods, and provided the opportunity for one-to-one individualized assessment. The Vida Health Spanish Diabetes Management Program provided all these opportunities. Particular care was taken in ensuring cultural congruency between the participants and the providers. Individuals in underserved medical communities are less likely to develop provider rapport and be offered evidence-based treatment interventions and achieve and sustain recommended outcomes when providers do not share the same language or do not have a thorough understanding of the cultural values of the patient [29]. In an ideal provider and patient setting, routine meetings with an educator, even at a distance and provided through an mHealth app, maintain active engagement and offer patients a high level of accountability for cocreated health goals [30]. The role of the provider has additionally been noted as a strong motivator in Hispanic/Latino culture, and a strong rapport with their providers may prompt participants to adopt new lifestyle habits and become better motivated to self-regulate their diabetes self-management behaviors [27,31,32].

### Strengths and Limitations

This study is not without its limitations. First, it was retrospective in nature; therefore, limited assumptions can be made about the causal relationship between the Vida Health Spanish Diabetes Management Program and the observed improvements in glycemic control. One component of diabetes management is the use of diabetes medications. While prescribing medications was outside the scope of the current program, the program content did provide education on the importance of medication adherence and management and encouraged regular visits with primary care physicians. Therefore, it is possible that diabetes medications contributed to the improvements in HbA_1c_ for some of the participants, particularly if they had been recently diagnosed with diabetes and initiated medications for the first time. Second, while great care was taken to ensure culturally appropriate matching of providers to patient needs, the Spanish-speaking community is complex and diverse, and cultural barriers may still have existed. Future work and future iterations of the Vida Health Spanish Diabetes Management Program will aim not only to expand Spanish-language cultural relevance but also to potentially develop other language-based diabetes health services. Third, this sample included participants from a United States–based payer organization, meaning all participants enrolled were employed and had some level of health insurance, which potentially limited the generalizability of our findings to individuals who may be unemployed or uninsured. Lastly, a number of individuals did not provide follow-up laboratory data, limiting the population sample size. Regardless, significant improvements in outcomes were observed among those who provided follow-up laboratory data.

### Conclusion

This study demonstrated that a comprehensively designed and culturally relevant diabetes treatment program that provided access to localized, culturally adapted, Spanish-language diabetes education and self-management content alongside frequent access to bilingual certified health coaches, registered dietitian nutritionists, and certified diabetes care and education specialists led to clinically significant improvements in glycemic control in Spanish-speaking individuals with type 2 diabetes. Future work is needed to understand barriers to intervention engagement among potential participants to enable access to available culturally adapted interventions such as the Vida Health Spanish Diabetes Management Program.

## Abbreviations

DSMES: diabetes self-management education and support
HbA_1c_: hemoglobin A_1c_
mHealth: mobile health

## Data Availability

The data sets analyzed during the current study are available from the corresponding author on reasonable request.
